# Construction and Tribological Properties of Biomimetic Cartilage-Lubricating Hydrogels

**DOI:** 10.3390/gels8070415

**Published:** 2022-07-01

**Authors:** Qiuyi Chen, Sa Liu, Zhongrun Yuan, Hai Yang, Renjian Xie, Li Ren

**Affiliations:** 1 School of Biomedical Science and Engineering, South China University of Technology, Guangzhou 510006, China; chenqy9409@163.com; 2 National Engineering Research Center for Tissue Restoration and Reconstruction, South China University of Technology, Guangzhou 510006, China; zryuan1997@163.com (Z.Y.); 202021021003@mail.scut.edu.cn (H.Y.); 3 School of Materials Science and Engineering, South China University of Technology, Guangzhou 510006, China; 4 School of Medical Information Engineering, Gannan Medical University, Ganzhou 341000, China; 5 Key Laboratory of Prevention and Treatment of Cardiovascular and Cerebrovascular Diseases, Ministry of Education, Gannan Medical University, Ganzhou 341000, China; 6 Key Laboratory of Biomaterials and Bio-Fabrication in Tissue Engineering of Jiangxi Province, Ganzhou 341000, China

**Keywords:** articular cartilage, osteoarthritis, cartilage replacement, polyvinyl alcohol, hydrogels, lubrication

## Abstract

Articular cartilage provides ultralow friction to maintain the physiological function of the knee joint, which arises from the hierarchical complex composed of hyaluronic acid, phospholipids, and lubricin, covering the cartilage surface as boundary lubrication layers. Cartilage-lubricating polymers (HA/PA and HA/PM) mimicking this complex have been demonstrated to restore the lubrication of cartilage via hydration lubrication, thus contributing to the treatment of early osteoarthritis (OA) in vivo. Here, biomimetic cartilage-lubricating hydrogels (HPX/PVA) were constructed by blending HA/PA and HA/PM (HPX) with polyvinyl alcohol (PVA) to improve the boundary lubrication and wear properties, so that the obtained hydrogels may offer a solution to the main drawbacks of PVA hydrogels used as cartilage implants. The HPX/PVA hydrogels exhibited good physicochemical and mechanical properties through hydrogen-bonding interactions, and showed lower friction and wear under the boundary lubrication and fluid film lubrication mechanisms, which remained when the hydrogels were rehydrated. Our strategy may provide new insights into exploring cartilage-inspired lubricating hydrogels.

## 1. Introduction

The daily movements of joints over a lifetime are maintained by the ultralow friction (friction coefficient ranging from 0.001 to 0.03 [1]) provided by articular cartilage at high pressures (even up to 10 to 20 MPa [2]) and low velocities. The low friction of articular cartilage is thought to be due to two main lubrication mechanisms, which are boundary lubrication and fluid film lubrication [3,4,5]. Specifically, the fascinating lubricity of cartilage has been attributed to fluid pressurization, supporting the majority of the load, and the boundary lubrication layers, which cover the surface of the cartilage by the self-assembly of molecules found in synovial fluid, including hyaluronan (HA), lubricin, and lipids [6,7,8,9]. When chondral injury occurs, the boundary layers of the outer cartilage surface are disrupted, which then causes the lubrication dysfunction of cartilage, resulting in increased friction and wear, which in turn leads to the onset of osteoarthritis (OA), whose major characteristic is the progressive degradation of articular cartilage [10,11,12], gradually causing joint pain and even disability in over 500 million people worldwide [13]. Therefore, treatment of the local chondral defects or partial joint repair is desperately needed to avoid the deterioration from OA. Current clinical treatments for articular cartilage defects mainly include microfracture, autologous chondrocyte implantation (ACI), and osteochondral autograft/allograft transplantation (OTC/OAT); however, these options cannot be used for large-area osteochondral defects, may lead to transplant rejection, and ignore the key lubrication properties of the articular cartilage [14,15,16].

With the aim of improving the treatment of articular cartilage defects, many attempts have been made to develop cartilage-like biomimetic materials. For decades, hydrogels, especially the polyvinyl alcohol (PVA) and PVA-based hydrogels, have been widely considered as an attractive alternative for cartilage replacement, with a great variety of performances similar to articular cartilage, such as biphasic nature, good biocompatibility, high water content, and mechanical robustness [17,18,19,20,21]. Despite these inherent biomimetic properties and their adaptability, the main drawbacks of PVA hydrogels compared to articular cartilage are their inferior friction and wear properties, particularly with regard to boundary lubrication [22,23]. Thus, lubrication properties are crucial to the PVA hydrogels when targeting cartilage replacement. Recently, several investigations have focused on the friction property of PVA hydrogels blended with highly hydrophilic polymers (such as polyethylene glycol, poly([2-(methacryloyloxy) ethyl] dimethyl-(3-sulfopropyl) ammonium hydroxide), poly(2-methacryloyloxyethyl phosphorylcholine), etc., which indicated that these polymers, acting as lubricants, could facilitate a reduction in the friction of hydrogels via hydration lubrication [24,25,26,27,28]. However, to date, there have been very few studies regarding simultaneously improving the friction and wear of PVA hydrogels to treat articular cartilage defects.

We previously reported cartilage-lubricating brush-like polymers (hyaluronic acid-graft- poly-2-acrylamide-2-methylpropanesulfonic acid sodium salt (HA/PA) and hyaluronic acid-graft- poly-2-methacryloyloxyethyl phosphoryl choline (HA/PM)) that mimicked the lubrication complexes (HA main chain and lubricin/lipids side chains [8,9]) and could effectively bind to the cartilage surface to form stable boundary layers in vitro and in vivo [6]. HA/PA and HA/PM were then found to lubricate and regenerate cartilage to heal early OA in vivo [6]. Based on these previous results, we postulate that the tribological performance and biomimetic nature of PVA hydrogels can be enhanced through the addition of HA/PA and HA/PM, which would provide an opportunity for introducing the boundary lubricating mechanism to the hydrogel. Therefore, the application of HA/PA and HA/PM to lubricate hydrogels will be crucial for cartilage implants.

To develop biomimetic cartilage-lubricating hydrogels, here we proposed to incorporate HA/PA and HA/PM (HPX) throughout the bulk of the PVA hydrogels to form HPX/PVA hydrogels using freeze-thaw cycles. This study characterized the physicochemical, mechanical, and tribological properties of the hydrogels to reveal their potential interconnections and influences, as well as cytotoxic properties. Further, this study also investigated hydrogen-bonding interactions between the polymers and the lubrication mechanism of the HPX/PVA hydrogels. The results showed that the HPX/PVA hydrogels exhibited better performance, exhibiting lower friction and wear than the PVA hydrogel alone, thus providing great applicational potential for partial cartilage repair.

## 2. Results and Discussion

### 2.1. Construction of the HPX/PVA Hydrogels

**Figure 1:**
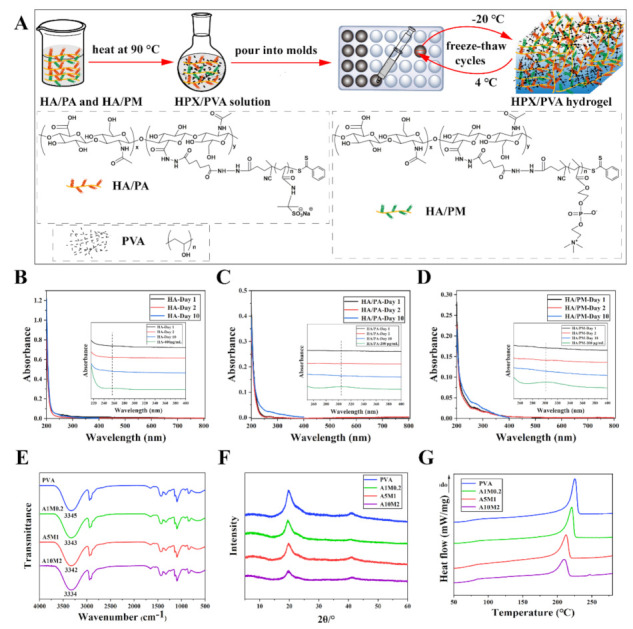
Construction and characterization of the HPX/PVA hydrogels. (**A**) Schematic illustrations of the preparation of the HPX/PVA hydrogels. UV-vis absorption spectra of extracts of the HA/PVA hydrogel (**B**); HA/PA/PVA hydrogel (**C**); HA/PM/PVA hydrogel (**D**). (**E**) The FTIR spectra of the PVA and HPX/PVA hydrogels. (**F**) The XRD patterns of the PVA and HPX/PVA hydrogels. (**G**) The DSC curves of the PVA and HPX/PVA hydrogels.

HA/PA and HA/PM (HPX) were prepared following our previous method [6]. Figure 1A shows schematic illustrations of the preparation of HPX/PVA hydrogels. The ratio of HA/PA to HA/PM in PVA was determined to be 5:1, according to our previous results; by changing the weight percent of HA/PA or HA/PM, four different hydrogels (named PVA, A1M0.2, A5M1, and A10M2) were prepared (Table S1). Element contents of the hydrogels were measured by elemental analysis (EA) and inductively coupled plasma mass spectrometry (ICP-MS), as depicted in Table 1. The contents of elements N, S, and P were increased with increasing HA/PA and HA/PM concentrations, and the measured values were close to the theoretical value (Table S2), which was sufficient to indicate the simultaneous presence of HA/PA and HA/PM within the PVA hydrogel. In other words, we have successfully prepared the HPX/PVA hydrogels.

To confirm that the HA/PA and HA/PM polymers were stably confined or encapsulated within the PVA hydrogel, the obtained HA/PA/PVA hydrogel and HA/PM/PVA hydrogel were firstly immersed in ultrapure water for 1, 2, and 10 days, and then the corresponding leach liquor was characterized by UV-vis absorption spectrum; no absorptions were observed between 200 and 800 nm (Figure 1B–D). As a control, the corresponding standard curves were established by measuring the UV spectra of HA, HA/PA, and HA/PM solutions with series known concentrations, and the results showed an absorption peak of the carbonyl group in HA at 256 nm and an absorption peak of the benzene ring in HA/PA or HA/PM at ~304 nm (Figure S1), which were in line with the previous reports [29,30]. These results demonstrated that HA/PA and HA/PM polymers were retained stably within the HPX/PVA hydrogels, which could be attributed to the fact that the movement of the HA, HA/PA, and HA/PM polymer chains were restricted due to the intermolecular entanglement, with partially crosslinked PVA chains [31]; additionally, the abundant hydrogen bonds between the HA/PA, HA/PM, and PVA chains further reinforce the intermolecular entanglement [6]. As a result, the stable and long-term retention of HA/PA and HA/PM in HPX/PVA hydrogels was achieved.

To verify the above results, we assessed the Fourier transform infrared spectroscopy (FTIR), X-ray diffraction (XRD), and differential scanning calorimetry (DSC) on the hydrogels. FTIR spectra demonstrated the characteristic stretching vibration of O-H at 3345 cm^−1^ of the PVA hydrogel gradually shifted towards a lower wavenumber as the concentration of HA/PA and HA/PM increased (Figure 1E), suggesting the formation and existence of hydrogen bonds between HA/PA, HA/PM, and PVA polymer chains [32,33]. The XRD results of the hydrogels showed that the intensity and area of 2θ at 19.6°, the typical diffraction peak belonging to PVA [34] was gradually weaker as compared to that of the PVA hydrogel (Figure 1F). As a result, the crystallinity of the hydrogels decreased (Table S3), which was attributed to the fact that the hydrogen bonds formed between HA/PA, HA/PM, and PVA consumed the O-H that determined the formation of PVA microcrystals [35]. Due to the intermolecular entanglement, PVA chains were also restricted, which in turn affected the glass transition temperature (Tg) [31], and as shown in DSC curves (Figure 1G), the Tg increased with increasing HA/PA and HA/PM concentrations (Table S4). Moreover, the crystallinity of the hydrogels was 51.8% (PVA), 45.1% (A1M0.2), 38.4% (A5M1), 31.3% (A10M2), respectively (Table S4). The addition of HA/PA and HA/PM resulted in a decrease in the melting temperature (Tm) and crystallinity of the HPX/PVA hydrogels, which indicated the hydrogen bonds formed between the PVA chains were reduced, thus affecting the microcrystallization of PVA [36]. In summary, the above results (FTIR, XRD, and DSC) implied the presence of hydrogen bonds between PVA (–OH) and HA/PA or HA/PM (–NH_2_ or –COOH), and the intermolecular entanglement formed between HA/PA and/or HA/PM chains and the PVA chains, which was essential for the persistence of HA/PA and HA/PM within the PVA hydrogel.

### 2.2. Physicochemical Properties of the HPX/PVA Hydrogels

**Figure 2:**
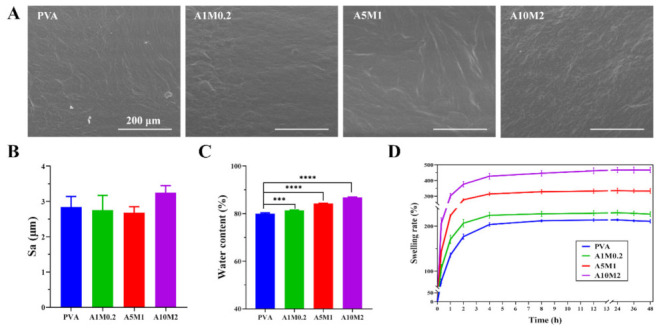
Physicochemical properties of the HPX/PVA hydrogels. (**A**) ESEM images of surface morphology of the PVA and HPX/PVA hydrogels; scale bar: 200 μm. (**B**) The surface roughness of PVA and HPX/PVA hydrogels. (**C**) Water content of PVA and HPX/PVA hydrogels. (**D**) Swelling ratio of PVA and HPX/PVA hydrogels. The error bars indicate the mean ± standard deviations from three independent experiments. Statistical analysis was performed using ordinary one-way analysis of variance (ANOVA) with Dunnett’s multiple comparisons (*** *p* < 0.001, **** *p* < 0.0001).

To mimic biological conditions, the surface morphology of the hydrogels in a fully swollen state was examined by environmental scanning electron microscope (ESEM), which showed small wrinkles, but which were largely flat, with barely changed surface morphology (Figure 2A). It was also clearly observed that there were no agglomerations of the HA/PA and HA/PM polymers, which revealed that HA/PA and HA/PM were well dispersed in the PVA matrix. Moreover, the surface roughness of the hydrogels was approximately 2.7 μm, with no significant difference (Figure 2B), which was consistent with the ESEM results. Based on these results, we hypothesized that the reduction in the friction coefficient of the HPX/PVA hydrogels was attributed to the lubrication effect of HA/PA and HA/PM, rather than the change in surface morphology.

**Figure 3:**
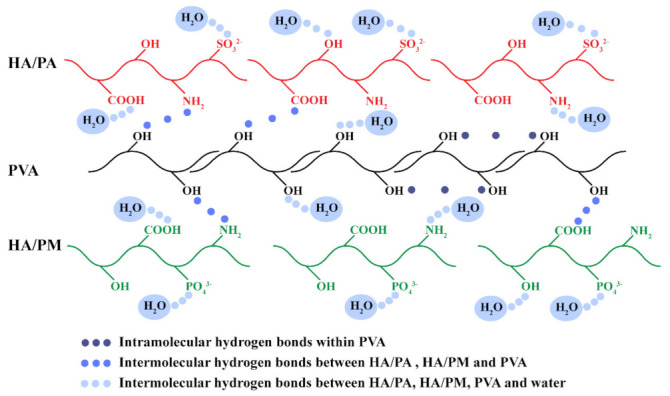
Schematic illustration of the formation and interactions of hydrogen bonds in the HPX/PVA hydrogels. Intramolecular hydrogen bonds were formed within PVA (–OH). Intermolecular hydrogen bonds were formed between HA/PA, HA/PM (–NH_2_ or –COOH), and PVA (–OH). Intermolecular hydrogen bonds were formed between HA/PA (–NH_2_, –COOH, –OH, –SO_3_^2−^), HA/PM (–NH_2_, –COOH, –OH, –PO_4_^3−^), PVA (–OH), and water.

Hydrogen bonds were formed, not only within and between polymer molecules, but also between polymer molecules and water molecules, resulting in water confined within the hydrogels [37]. Water content is a material property that represents the water retention capacity of the hydrogels. The water content of the hydrogels increased significantly in the order of PVA (80.0%), A1M0.2 (81.4%), A5M1 (84.2%), and A10M2 (87.8%) (Figure 2C), which was attributed to the high hydrophilicity of HA/PA and HA/PM [6], and was close to the high water content, ranging from 65% to 85%, of normal articular cartilage [38]. In addition, when swelling equilibrium was reached after 12 h, the swelling rates of the hydrogels successively increased significantly, at 213% (PVA), 228% (A1M0.2), 333% (A5M1), and 462% (A10M2) (Figure 2D), which were comparable to that of natural articular cartilage, ranging from 200% to 400% [39]. As the water gradually penetrated the PVA hydrogel network, it formed bound water by hydrogen bonding with the polar groups (–OH) until it reached saturation values [37]. The addition of hydrophilic groups of HA/PA and HA/PM, such as –NH_2_, –COOH, –OH, –SO_3_^2−^, and –PO_4_^3−^ [6], led to the formation of a small number of hydrogen bonds with water, thus enhancing the hydrophilicity of the HPX/PVA hydrogels, as shown in Figure 3. Additionally, the contact angles of the hydrogels ranged from 17 to 19° (Figure S2), which further confirmed the high hydrophilicity of the hydrogels. In short, these results demonstrated that incorporating HA/PA and HA/PM within the PVA hydrogel resulted in improved hydrophilicity and did not alter the surface of the hydrogels. More importantly, the higher water content and swelling rate of the HPX/PVA hydrogels exhibited a combination of more robust liquid retention and absorption capability, allowing for better lubrication applications [40]. It was interesting to note that the formation and interactions of hydrogen bonds within PVA, between HA/PA, HA/PM, and PVA, and between HA/PA, HA/PM, PVA, and water contributed to the persistence of HA/PA and HA/PM in the HPX/PVA hydrogels, thus improving the water content, swelling rate, and the mechanical and lubrication properties of the HPX/PVA hydrogels, as schematically indicated in Figure 3.

### 2.3. Mechanical Properties of the HPX/PVA Hydrogels

**Figure 4:**
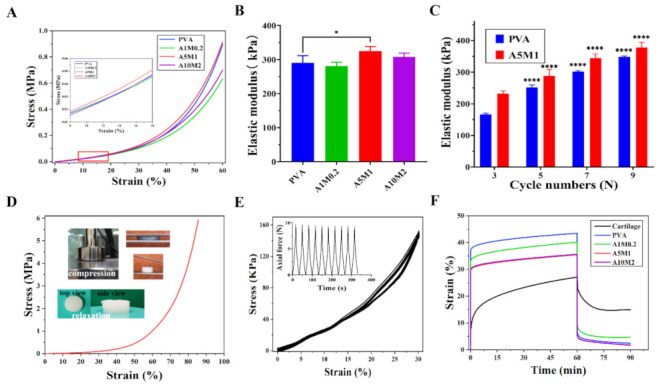
Mechanical properties of the HPX/PVA hydrogels. (**A**) The compressive stress–strain curves of the PVA and HPX/PVA hydrogels.; illustration from the linear regime, with the strain range of 8–18%. (**B**) The compressive moduli of the PVA and HPX/PVA hydrogels. (**C**) The effect of the number of freeze-thaw cycles on the compressive moduli of the PVA and A5M1 hydrogels. (**D**) The compressive stress–strain curve of the A5M1 hydrogel compressed to 85% strain; illustration of compression and relaxation of the A5M1 hydrogel. (**E**) The cyclic compressive stress–strain curves of the A5M1 hydrogel; illustration of time and axial force in the cyclic compressive test. (**F**) The creep curves of the PVA and HPX/PVA hydrogels versus porcine articular cartilage. The error bars indicate the mean ± standard deviations from at least three independent experiments. Statistical analysis was performed using ANOVA with Dunnett’s multiple comparisons (* *p* < 0.05, **** *p* < 0.0001).

Compression tests were conducted to understand the mechanical properties of the HPX/PVA hydrogels. Figure 4A shows the stress–strain curves of the hydrogels, and the inset shows the linear regime, with strains ranging from 8% to 18%. The compressive moduli of the hydrogels were 290.6 KPa (PVA), 281.3 KPa (A1M0.2), 325.3 KPa (A5M1), and 307.9 KPa (A10M2), which did not decline significantly with the inclusion of HA/PA and HA/PM (Figure 4B). Nevertheless, the compressive moduli of the A5M1 and A10M2 hydrogels were within the range of those of native cartilage, that is, from 300 KPa to 800 KPa [41]. It is worth noting that no clear trend of the results was observed when the concentrations of HA/PA and HA/PM increased, which might be due to the competition between the restricted movement of PVA chains themselves (see Tg results) and the enhanced multiple hydrogen bonding interactions (see Figure 3). From this, the A5M1 hydrogel achieved the maximum compressive modulus. Sequentially, we researched the effect of the number of freeze-thaw cycles on the compressive moduli of the PVA and A5M1 hydrogels (Figure 4C). The compression moduli of the hydrogels grew as the number of freeze-thaw cycles increased. During the freeze-thaw of PVA hydrogel, the physical crosslinking points within the PVA chains continuously increased through hydrogen bonding interactions, which then improved the mechanical properties [42]. Remarkably, the compression modulus of the A5M1 hydrogel was superior to that of the PVA hydrogel; therefore, we considered that this could be the creation of more and stronger hydrogen bonds between HA/PA, HA/PM, and PVA. The A5M1 hydrogel was not destroyed after being compressed to 85% strain and recovered after immersion in water (Figure 4D). Moreover, a cyclic compression test of the A5M1 hydrogel showed minimal change in deformation over 10 cycles at 30% strain (Figure 4E). These results revealed that the HPX/PVA hydrogels had sufficiently high mechanical stability and high elasticity, with rapid recovery.

To further explore the mechanical matching performance of the hydrogels versus native articular cartilage, the viscoelastic properties were estimated using creep tests. The creep curves of the PVA hydrogel were obtained at different pressures (stress ranging from 0.01 to 0.15 MPa) for 60 min and with the load removed for 30 min (Figure S3). The creep curves of the hydrogels and porcine cartilage at a constant compressive stress of 0.1 MPa (maximum recovery rate) are shown in Figure 4F. The initial creep strains of the hydrogels instantaneously reached after compression were higher than those of cartilage, as were the final creep strains, with an even larger creep deformation than that of the cartilage. The smaller creep deformations of the hydrogels were associated with a decrease in the molecular movement and relaxation of the polymer chains under the action of hydrogen bonding [43], along with interstitial fluids within the polymer network [44]. The cartilage exhibited a larger creep flow because of the layered structure of the cartilage, composed of proteoglycans and a collagen network, the molecular movement through reversible interactions, and the osmotic pressure of cartilage tissues [45,46,47]. Hence, the intrinsic structural differences between synthetic hydrogels and native articular cartilage pointed to the hydrogels exhibiting more elastic responses, whereas cartilage exhibited a more viscous nature [43]. Overall, the instantaneous deformations and creep deformations of the HPX/PVA hydrogels decreased with increasing HA/PA and HA/PM concentrations, as a result of increased hydrogen bonds and interstitial fluid, which corresponded to load-bearing function (compressive modulus in Figure 4B) and water retention capacity (water content in Figure 2C). Similarly, the phenomenon of smaller initial recovery and final recovery strains for the hydrogels than for the cartilage was observed during recovery, with the hydrogels recovering much faster than cartilage. It follows that the viscoelastic properties of the HPX/PVA hydrogels approached those of the native articular cartilage relative to the PVA hydrogel, which benefits from the biomimetic nature of HA/PA and HA/PM. Recovery rates for the PVA, A5M1, and A10M2 hydrogels reached 95% after 30 min of load removal, while cartilage took a much longer time to fully recover. Altogether, the HPX/PVA hydrogels had good mechanical properties to withstand loads, cushion pressure, and absorb shocks.

### 2.4. Friction and Wear Properties of the HPX/PVA Hydrogels

**Figure 5:**
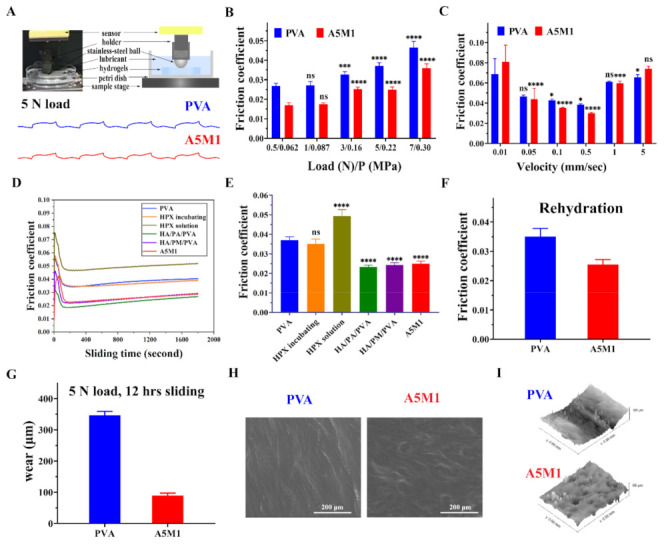
Friction and wear properties of the HPX/PVA hydrogels. (**A**) The UMT tribometer configuration and representative curves recorded directly for the PVA and A5M1 hydrogels. (**B**) The variation of μ at a series of loads for the PVA and A5M1 hydrogels under velocity of 0.5 mm/s. Various loads correspond to different contact pressure P. (**C**) The variation of μ as a function of the sliding velocity for the PVA and A5M1 hydrogels under load of 5 N. (**D**) The friction curves of the hydrogels versus sliding time under 5 N load with 0.5 mm/s. Sliding friction between PVA hydrogel (blue), between PVA hydrogel after incubation of HA/PA and HA/PM solutions (orange), followed by washing, after 30 min of sliding, and between PVA hydrogel immersed in HA/PA and HA/PM solutions (olive green), compared with HA/PA/PVA hydrogel (green), HA/PM/PVA hydrogel (purple), A5M1 hydrogel (red) immersed in PBS. (**E**) The corresponding friction coefficient of the hydrogels. (**F**) The friction coefficient of the A5M1 and PVA hydrogels after rehydration. (**G**) Wear of the PVA and A5M1 hydrogels after 12 h of sliding at 5 N load. (**H**) The corresponding ESEM images of the worn PVA and A5M1 hydrogels (scale bar: 200 μm). (**I**) The corresponding 3D morphology maps of the worn PVA and A5M1 hydrogels. Error bars indicate SD from at least three independent measurements. Statistical analysis was performed using ANOVA with Dunnett’s multiple comparisons (* *p* < 0.05, *** *p* < 0.001, **** *p* < 0.0001, ns: not significant.).

Competent biomimetic cartilage-lubricating hydrogels should possess good lubrication properties. The hydrogels were prepared by 7 freeze-thaw cycles, as the friction coefficient dropped to the saturation point after 7 freeze-thaw cycles (Figure S4). All factors, including water content, compressive modulus, and friction coefficients, were considered collectively to determine the subsequent tribological properties of the A5M1 hydrogel. Figure 5A shows the tribometer configuration and representative curves recorded directly, which determined the friction force and friction coefficient μ.

**Figure 6:**
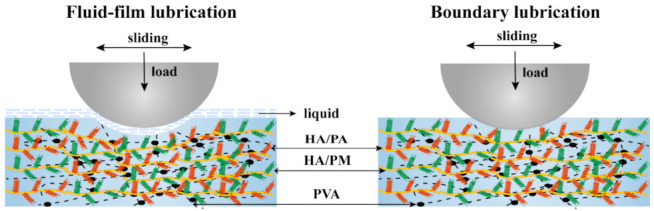
Schematic diagrams of the lubrication mechanism of the HPX/PVA hydrogels. Mixed lubrication due to the coexistence of boundary lubrication and fluid-film lubrication.

Figure 5B showed the variation of μ with an applied load for the PVA and A5M1 hydrogels. For the PVA hydrogel, μ ranged from 0.026, at lower loads, to 0.046, at higher loads, whereas for the A5M1 hydrogel, the μ was 0.017 to 0.036. We observed a 36% to 23% reduction in μ (from low to high loading) for the A5M1 hydrogel relative to the PVA hydrogel, specifically, a 32% reduction at 5 N loads. These results could mainly be attributed to the hydration lubrication of HA/PA and HA/PM, which were surrounded by abundant charged groups (SO_3_^2−^, PO_4_^3−^ and N^+^(CH_3_)_3_), with high hydration ability to form hydration shells [6,48]. We also considered that the compression modulus of the A5M1 hydrogel was stronger than that of the PVA hydrogel, which allowed the A5M1 hydrogel to support a much higher load (see Figure 4B) [49,50]. In addition, the hydrogels exhibited higher μ at higher loads, which was due to the fact that the fluid film was squeezed out, leading to larger deformation at high pressures [40,51]. Figure 5C showed that μ of the PVA and A5M1 hydrogels tended to decrease and then increase with speed over three orders of magnitude. The A5M1 hydrogel had the same lower μ as the PVA hydrogel due to the hydration layers arising from HA/PA and HA/PM. The friction coefficient of the A5M1 hydrogel indicated the signature of mixed lubrication due to the coexistence of boundary lubrication and fluid-film lubrication [38,52], as indicated schematically in Figure 6.

The lubrication using externally applied HA/PA and HA/PM was far less effective than the internally incorporated HA/PA and HA/PM, which determined the potential for lubricating hydrogels in practical applications of cartilage replacement. Figure 5D shows the friction profile of the hydrogels as a function of sliding time, where the stability of the friction was observed. Figure 5E shows the corresponding μ. For the case of HA/PA and/or HA/PM incorporated PVA hydrogels sliding in PBS (μ ≈ 0.025), μ was minimal and stabilized with sliding time, because there were sufficient internal lubricating polymers. When the PVA hydrogel was incubated in HA/PA and HA/PM solution overnight, then submitted to sliding in PBS (μ ≈ 0.035), μ increased, but was the same as for the PVA hydrogel, due to little adsorption of HA/PA and HA/PM at the friction interface [3]. However, the lubrication of the PVA hydrogel deteriorated rapidly when sliding in HA/PA and HA/PM solutions (μ ≈ 0.049). These results indicated that incorporating HA/PA and/or HA/PM within the PVA hydrogels resulted in much lower friction and more effective lubrication than in the situation where the PVA hydrogels were externally exposed to HA/PA and HA/PM solutions. Thus, HA/PA and HA/PM incorporated within PVA hydrogels formed stable hydrated lubrication layers. After the A5M1 and PVA hydrogels were completely dried and fully rehydrated, μ still remained at its original low value, and the lubrication was again maintained (Figure 5F). The robustness of dehydration and rehydration was beneficial for the storage of hydrogels in particular applications, which again suggested that HA/PA and HA/PM were robustly incorporated in the PVA hydrogels.

Finally, the wear and surface damage of the A5M1 hydrogel were substantially reduced, as noted by comparing the wear of the PVA hydrogel and A5M1 hydrogel after 12 h of sliding under a 5 N load, as shown in Figure 5G–I. Under these conditions, the surface wear of the A5M1 hydrogels was ~90 μm, while the wear of the PVA hydrogel was ~347 μm, showing an approximately fourfold reduction (Figure 5G). This suggested that the A5M1 hydrogel could resist wear from repeated sliding cycles for a long period of time. The effect of surface wear and damage on the A5M1 and PVA hydrogels is manifested by the ESEM images (Figure 5H) and their corresponding 3D morphology maps (Figure 5I). We did not observe any obvious surface tear or damage on the hydrogels. Scratches were observed on the highly worn PVA hydrogel, but not on the A5M1 hydrogel. Of particular interest was the appearance of some bleb shape in the A5M1 hydrogel; thus, we conjectured that this was the result of HA/PA and HA/PM being exposed to resistance to wear. All in all, friction, wear, and surface damage were all reduced by blending HA/PA and HA/PM. Consequently, the application of HA/PA and HA/PM to cartilage implants will be important for partial cartilage repair. Collectively, our study offers a solution to the main drawbacks of the inferior friction and wear properties of PVA hydrogels used as cartilage implants, which sheds new light on exploring the introduction of boundary lubrication mechanisms into PVA hydrogels.

### 2.5. Cytotoxicity Properties of the HPX/PVA Hydrogels

**Figure 7:**
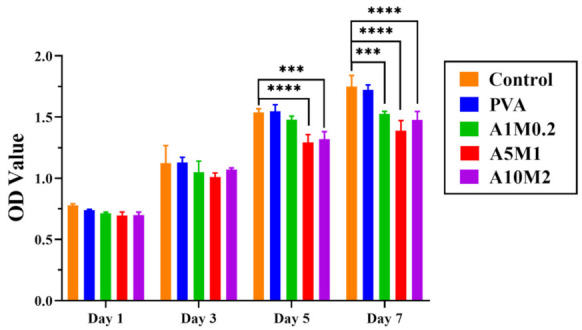
The Cytotoxicity of extracts of PVA and HPX/PVA hydrogels. The error bars indicate the mean ± standard deviations from three independent experiments. Statistical analysis was performed using two-way ANOVA with Dunnett’s multiple comparisons (*** *p* < 0.001, **** *p* < 0.0001).

The cytotoxicity of extracts of PVA and HPX/PVA hydrogels was investigated by CCK-8 assay. The survival rate of chondrocytes in the groups of HPX/PVA hydrogels maintained above 80% after being cultured for 7 days (Figure 7), which showed that the HPX/PVA hydrogels had good cytocompatibility and laid the foundation for in vivo experiments.

## 3. Conclusions

In summary, incorporating biomimetic lubricants (HA/PA and HA/PM) within the PVA hydrogels provided a simple route to successfully construct biomimetic cartilage-lubricating hydrogels, the HPX/PVA hydrogels, which showed high water content and good mechanical properties via hydrogen-bonding interactions. More importantly, the HPX/PVA hydrogels exhibited lower friction and wear under hydration lubrication, which indicated the signature of mixed lubrication. Therefore, our study may offer an excellent platform for creating lubricating hydrogels with low friction and wear. Further studies on the visual distribution and lubrication processes of HA/PA and HA/PM in the hydrogels, as well as the biocompatibility of the HPX/PVA hydrogels, are needed to demonstrate their efficiency for cartilage replacement.

## 4. Materials and Methods

### 4.1. Materials

Polyvinyl alcohol (PVA, 99+% hydrolyzed, Mw 89,000–98,000), 2-acrylamido-2-methylpropanesulfonic acid (AMPS), 4,4′-azobis (4-cyanovaleric acid) (ACVA), N-ethyl-N-(3-(dimethylamino) propyl) carbodiimide (EDC), N-hydroxysuccinimide (NHS), adipic dihydrazide (ADH), and 4-cyanopentanoic acid dithiobenzoate (CTP) were purchased from Sigma-Aldrich. Sodium hyaluronate (Mw 1.0–1.5 × 10^3^ kDa) was obtained from the Shanghai Yuanye Biological Technology. 2-Methacryloyloxy ethyl phosphorylcholine (MPC) was obtained from Jenkem Technology.

### 4.2. Preparation of the HPX/PVA Hydrogels

HA/PA and HA/PM were prepared according to the literature [6] (Figure S5). HA/PA and HA/PM were dissolved in ultrapure water to prepare the solutions, with different concentrations, at a ratio of 5:1. A total of 15 wt% of PVA was added and then mixed with HA/PA and HA/PM solutions for 2 h at 90 °C, with mechanical agitation. After air bubbles were removed, the mixtures were poured into molds. The hydrogels were prepared by freezing them at −20 °C for 8 h and thawing at 4 °C for 16 h, with 7 freeze-thaw cycles. In brief, we referred to the corresponding hydrogels as PVA, A1M0.2, A5M1, and A10M2 hydrogels, according to different concentrations (mg/mL), respectively (Table S1). Collectively, three composite hydrogels were referred to as HPX/PVA hydrogels.

### 4.3. Characterization of Hydrogels

#### 4.3.1. Elemental Analyses of Hydrogels

Elemental analyses (C, H, N, and S) of the lyophilized hydrogels were performed on a Vario EL Cube elemental analyzer (EA); P was measured using an inductively coupled plasma mass spectrometer (ICP-MS, iCAP RQ, Thermo Scientific, Waltham, MA, USA).

#### 4.3.2. UV-Visible Spectroscopy of Hydrogel Extracts

Absorbance spectra were recorded on a UV-visible spectrophotometer (UV-2600, Shimadzu). Different concentrations of HA, HA/PA, and HA/PM solutions were prepared using ultrapure water. The concentrations were 100, 200, 400, 600, 800, and 1000 μg/mL, respectively. The absorbances of HA, HA/PA, and HA/PM solutions were recorded at a wavelength of maximal absorbance of 256, 304, and 301 nm, respectively. The standard curves of HA, HA/PA, and HA/PM were obtained by a linear fit, according to the Beer–Lambert law (Figure S1). The HA/PVA, HA/PA/PVA, and HA/PM/PVA hydrogels were immersed in ultrapure water and refilled with an equal volume at the time points of day 1, 2, and 10, immediately after taking 3 mL of solution. The absorbance of each sample was measured at predetermined time points using a spectrophotometer.

#### 4.3.3. Reflection Fourier Transform Infrared of Hydrogels

The FTIR spectra of the lyophilized hydrogels were produced using a Bruker Vector 33 FTIR spectrometer in a range of 4000–500 cm^−1^ with 4 cm^−1^ per step width.

#### 4.3.4. X-ray Diffraction of Hydrogels

X-ray diffraction (XRD) characterization was conducted with a PANalytical Empyrean X-ray diffractometer (Cu Kα = 1.54 Å). Data collection was carried out over a scanning range of 5 to 60°, with a scan step size of 0.02° and a scan speed of 5°/min. The test samples were lyophilized hydrogels.

#### 4.3.5. Differential Scanning Calorimetry of Hydrogels

The glass transition temperature (Tg) and the melting temperatures (Tm) of the hydrogels were measured by differential scanning calorimetry (NETZSCH, DSC 214 Polyma) in order to determine the enthalpy of fusion and crystallinity. Heating scans were recorded in the range of 25 to 300 °C, at a scan rate of 10 K/min, in a nitrogen atmosphere. DSC curves of the heat flow with respect to the temperature were obtained at the second heating process.

#### 4.3.6. Surface Morphology and Roughness

The surface morphology of wet hydrogels was characterized using a FEI Quanta 200 environmental scanning electron microscope (ESEM) at 20 kV. The surface roughness was measured using a 3D optical profilometer (RTEC, UP Dual Model, San Jose, CA, USA) with a ×20 objective. The images were processed with the software Gwyddion to calculate arithmetic average roughness (Ra).

#### 4.3.7. Water Content, Swelling Ratio, and Contact Angle

The wet weight (W_0_) of the hydrogels was determined during swelling equilibrium in deionized water. After the samples were lyophilized, the dry weight (W_d_) of the hydrogels was measured. Then, the samples were immersed in deionized water. The sample mass (W_s_) was recorded systematically when the samples were removed from water; water content (%) = (W_0_ − W_d_)/W_0_ × 100%; swelling ratio (%) = (W_s_ − W_d_)/W_d_ × 100%. Each measurement was performed with three parallel samples. The contact angles of the wet hydrogel surface were measured using a contact angle goniometer (DSA25, Kruss, Germany).

### 4.4. Compression Mechanical Testing

The compressive stress–strain measurements were performed using a universal testing machine with a 50 N sensor (Instron 5967, Norwood, MA, USA). The compressive samples were prepared at 10 mm in diameter and 5 mm in height. The compressive strain rate was 5 mm/min up to 60% or even 85% of strain. The compressive moduli were calculated within the strain range of 8 to 18%. The cyclic compression test was performed using a tension and torsion composite testing machine (SASTest, UTM2102, Beijing, China), with a 100 N sensor, under 30% strain for 10 cycles.

### 4.5. Creep Testing

Creep measurements were performed using a dynamic mechanical analyzer (DMA Q800, TA Instruments, New Castle, DE, USA) to compare the viscoelastic behavior of the hydrogels and porcine cartilage. The creep samples were prepared in the same way as the compression samples above. Creep curves were recorded along with time to assess the instantaneous strain and creep deformation, while a constant stress (0.1 MPa) was applied for 1 h, and then recovery curves were recorded during unloading for 30 min. Prior to this, creep measurements for the PVA hydrogels were performed under different stresses, ranging from 0.01 to 0.15 MPa.

### 4.6. Friction and Wear Measurements

Friction measurements were carried out using a UMT-2 tribometer (Bruker, Camarillo, CA, USA). Measurements in all cases were carried out under PBS, unless otherwise stated. The friction force Fs between the hydrogels and the polished stainless-steel spherical head (diameter 9 mm) was measured over a 30 min sliding time under the reciprocating motion mode at different sliding velocities and loads. Sliding velocities were in the range of 0.01 to 5 mm/s. Loads of F_n_ ranging from 0.5 N to 7 N corresponded to different mean pressures P = (F_n_/A) over the contact area A. Friction coefficient µ was evaluated at each load as µ = F_s_/F_n_. Sliding friction was compared between PVA hydrogels, between PVA hydrogels after incubation of HA/PA and HA/PM solution followed by washing, and between PVA hydrogels immersed in HA/PA and HA/PM solution, compared with HA/PA/PVA, HA/PM/PVA, and A5M1 hydrogels over 30 min sliding time at sliding velocities of 0.5 mm/s and loads of 5 N. The same test was performed for the rehydrated PVA and A5M1 hydrogels. The surface damage was imaged with ESEM 3D morphology maps, and the wear of the PVA and A5M1 hydrogels was characterized by a 3D optical profilometer after sliding for 12 h.

### 4.7. Cytocompatibility

To evaluated the cytocompatibility in vitro of the hydrogels, the cytotoxicity was measured using a Cell Counting Kit-8 assay. Chondrocytes were harvested from the patellar cartilage of rabbits, according to the method report previously [6]. The extract of the hydrogels were used according to the national standard GB/T 16886.12–2005. Chondrocytes were seeded at a concentration of 3.0 × 10^3^ cells per well^−1^ in 96-well plates with 100 μL of media, and then the extracts were added into each well. The chondrocytes were incubated with CCK-8 working solution for 4 h at 37 °C and 5% CO_2_ after culturing for 1, 3, 5, and 7 days. Then the absorbance was measured at 450 nm using a microplate reader (Type3001, Thermo Fisher Scientific, Waltham, MA, USA).

### 4.8. Statistical Analysis

Quantitative data are expressed as the mean ± standard deviation based on at least three independent experiments. Significant differences were identified by analysis of variance (ANOVA) for independent samples. * *p* < 0.05, ** *p* < 0.01, *** *p* < 0.001, **** *p* < 0.0001 were accepted as statistically significant, and nonsignificant meant that there was no statistically significant difference.

## Figures and Tables

**Table 1:** The element contents of the PVA and HPX/PVA hydrogels.

Elements	PVA	A1M0.2	A5M1	A10M2
C%	47.55	48.55	49.08	47.42
H%	6.84	8.14	8.27	8.15
N%	0	0.02	0.21	0.33
S%	0	0.01	0.12	0.22
P%	0	0.01	0.03	0.04

## Data Availability

All data generated from the study are available in the manuscript or the Supplementary Materials.

## Supplementary Materials

The following supporting information can be downloaded at: https://www.mdpi.com/article/10.3390/gels8070415/s1, Figure S1: UV spectra and standard curves of HA, HA/PA, and HA/PM of different concentrations; Figure S2: Contact angle of the PVA and HPX/PVA hydrogels; Figure S3: Creep curves of the PVA hydrogel under different pressures; Figure S4: Friction coefficient of the PVA and HPX/PVA hydrogels for 7 and 9 freeze-thaw cycles; Figure S5: Synthetic route for the HA/PA **(A**), and HA/PM **(B);** Supplementary Table S1: The composition of the PVA and HPX/PVA hydrogels; Table S2: The theoretical element contents of the PVA and HPX/PVA hydrogels. Table S3: Crystallinity from XRD patterns of the PVA and HPX/PVA hydrogels; Table S4: Glass transition temperature (Tg), melting temperature (Tm), melting enthalpy (ΔHm), and crystallinity from DSC curves of the PVA and HPX/PVA hydrogels.
