# Supplementary material for: Construction and Tribological Properties of Biomimetic Cartilage-Lubricating Hydrogels
Source: Gels. 2022 Jul 1;8(7):415. doi: 10.3390/gels8070415 (PMC9319379; doi:10.3390/gels8070415)
Supplement: Supplementary file 1 [file gels-08-00415-s001.zip › gels-1779248-supplementary.pdf]

Construction and Tribological Properties of Biomimetic Cartilage-lubricating Hydrogels

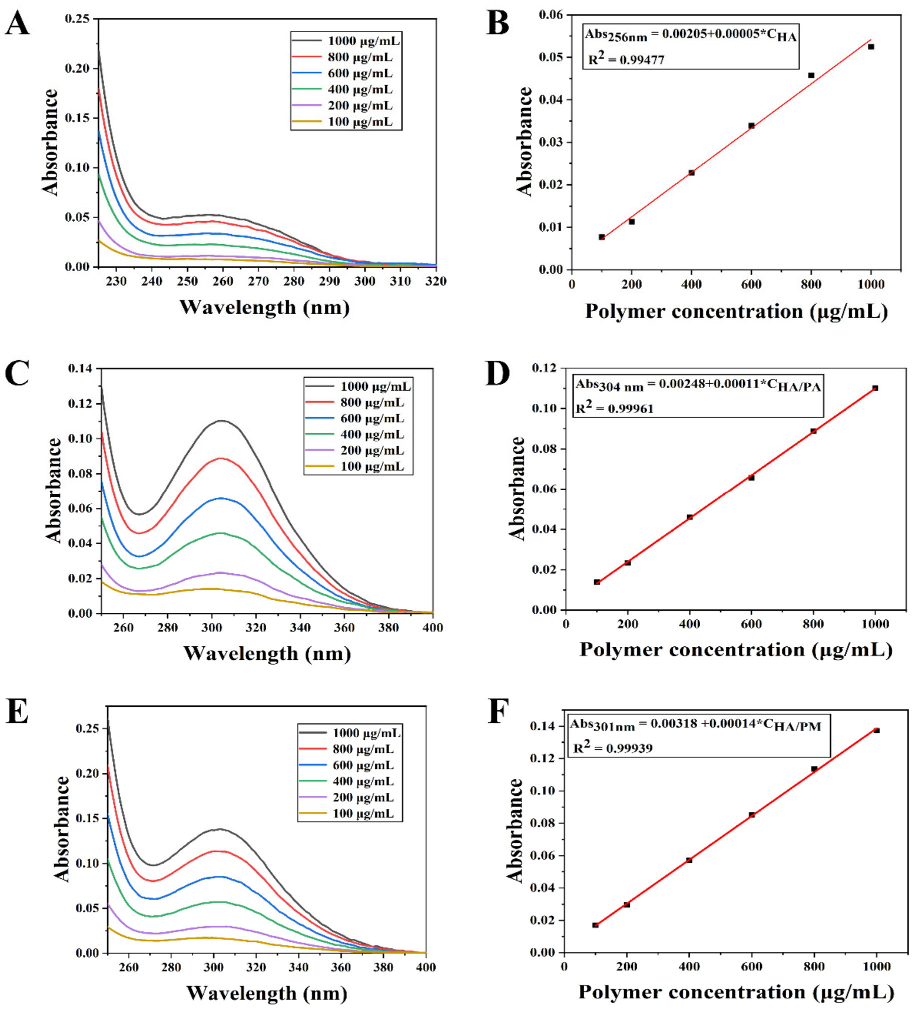

Figure S1. UV spectra and standard curves of HA, HA/PA and HA/PM of different concentrations. **(A)** UV spectra of HA. **(B)** Standard curves of HA. **(C)** UV spectra of HA/PA. **(D)** Standard curves of HA/PA. **(E)**UV spectra of HA/PM. **(F)** Standard curves of HA/PM.

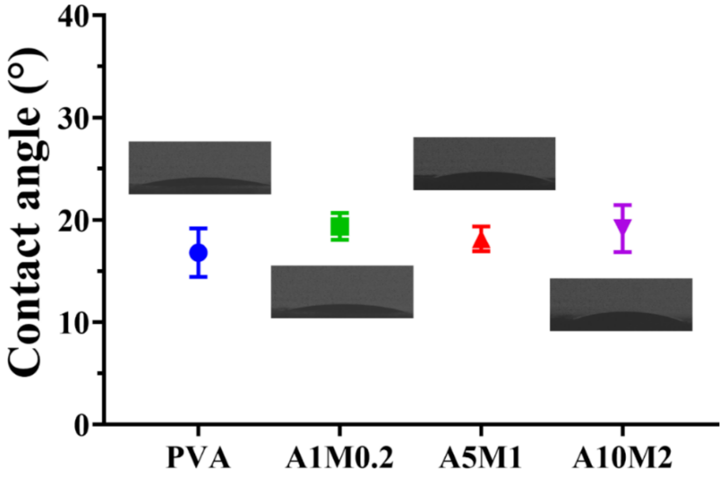

Figure S2. Contact angle of the PVA and HPX/PVA hydrogels.

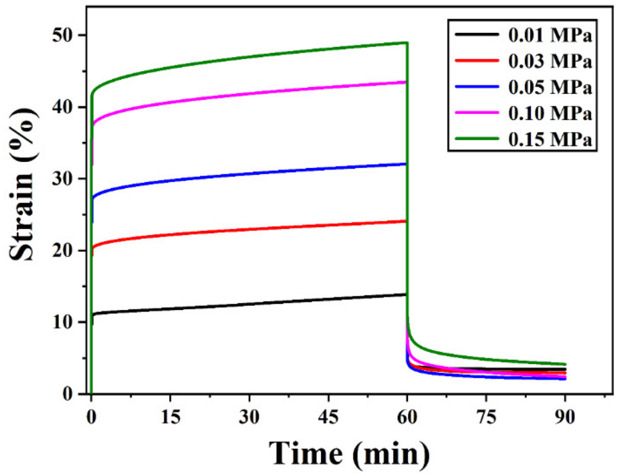

Figure S3. Creep curves of the PVA hydrogel under different pressures.

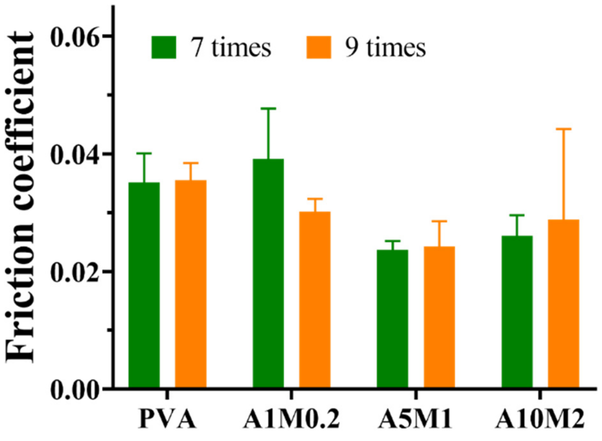

Figure. S4. Friction coefficient of the PVA and HPX/PVA hydrogels for 7 and 9 freeze-thaw cycles.

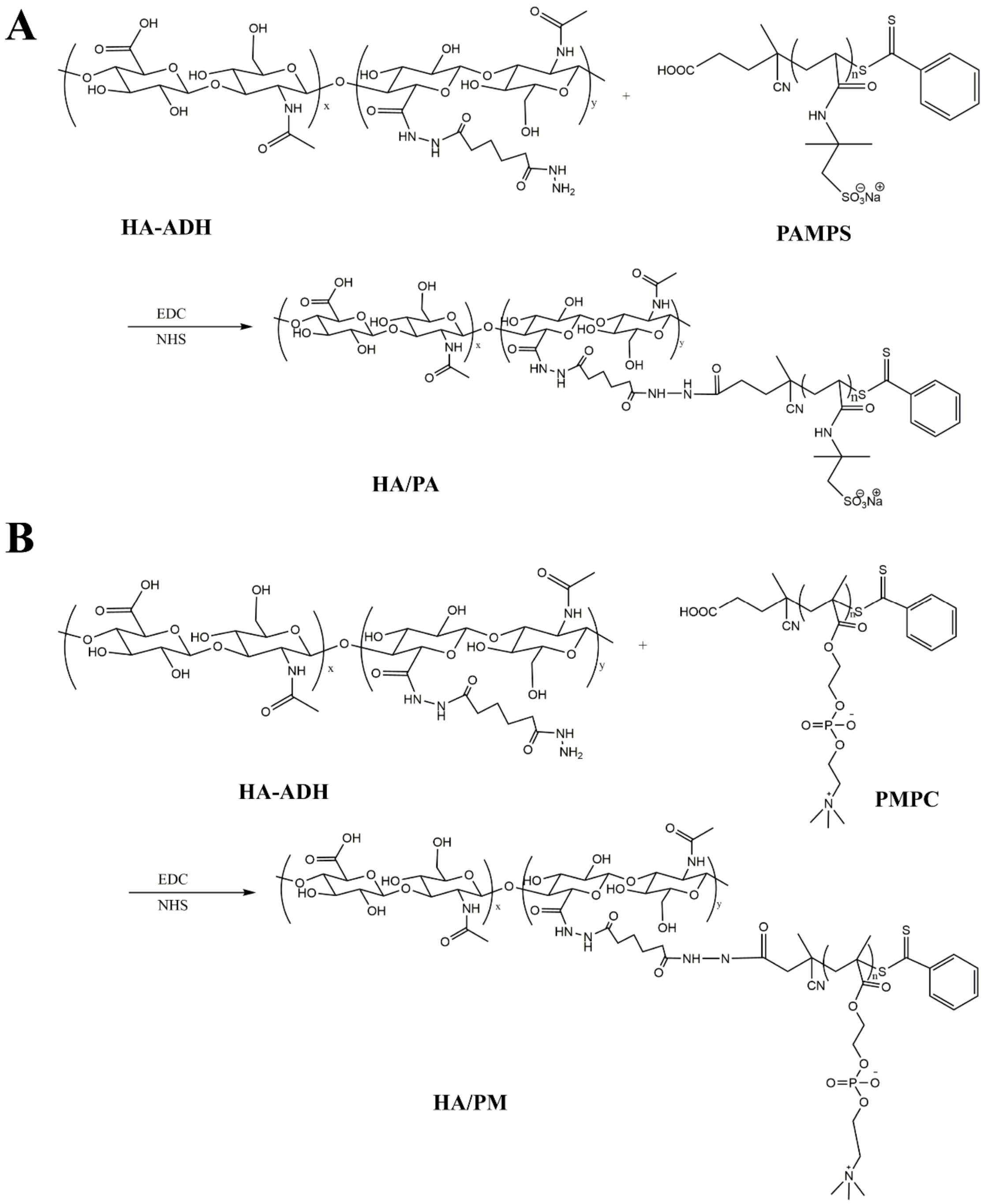

Figure S5. Synthetic route for the HA/PA **(A**), and HA/PM **(B)**.

Table S1. The composition of the PVA and HPX/PVA hydrogels.

Sample	PVA (wt%)	HPA (wt%)	HPM (wt%)
PVA	15	0	0
A1M0.2	15	0.085	0.017
A5M1	15	0.425	0.085
A10M2	15	0.850	0.17

Table S2. The theoretical element contents of the PVA and HPX/PVA hydrogels.

Elements	PVA	A1M0.2	A5M1	A10M2
**N%**	0	0.05	0.2	0.47
**S%**	0	0.09	0.43	0.84
**P%**	0	0.01	0.05	0.11

Table S3. Crystallinity from XRD patterns of the PVA and HPX/PVA hydrogels. Crystallinity for the PVA hydrogel was referred as 100%, and crystallinity of HPX/PVA hydrogels were normalized to that of the PVA hydrogel.

Sample	Height	Area	Crystallinity%
PVA	856	74927	100
A1M0.2	621	54523	72
A5M1	593	50224	69
A10M2	313	27739	36

Table S4. Glass transition temperature (Tg), the melting temperature (Tm), melting enthalpy (ΔHm) and crystallinity from DSC curves of the PVA and HPX/PVA hydrogels. ΔHm was calculated by integrating the peak areas of melting peaks. ΔHm* of PVA is 138.6 J/g. Crystallinity Xc% = ΔHm/ΔHm*.

Sample	Tg (℃)	Tm (℃)	ΔHm (J/g)	Xc%
PVA	73	225	71.8	51.8
A1M0.2	77	220	62.5	45.1
A5M1	75	212	53.3	38.4
A10M2	81	210	43.4	31.3
